# Supplementary material for: Proteomic and Global DNA Methylation Modulation in Lipid Metabolism Disorders with a Marine-Derived Bioproduct
Source: Biology (Basel). 2023 Jun 2;12(6):806. doi: 10.3390/biology12060806 (PMC10295662; doi:10.3390/biology12060806)
Supplement: Supplementary file 1 [file biology-12-00806-s001.zip › Suppl. Fig. S1_biology-2302663_resub.pdf]

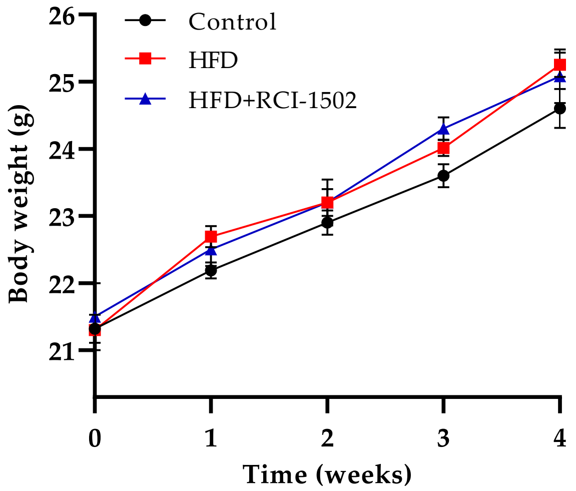


**Suppl. Fig. S1.** Body weights of mice over four weeks on three different diets: a normal diet (control), a high-fat diet (HFD, corn oil), and an HFD supplemented with RCI-1502 (HFD + RCI-1502). At the start of week 4, the mice in Group C were switched to the HFD + RCI-1502 diet for seven days. For those (Group C) mice, food pellets containing only corn-oil were mixed with RCI-1502 (2.53–5.06 mg/day/mouse), and recompacted into pellets. The bodyweights of the mice in each group were recorded at the end of each week. Data were analyzed with a Kruskal-Wallis test with Dunn's *post hoc* multiple comparisons; $^{*}p < 0.05$ was considered statistically significant.
