# Supplementary material for: Proteomic and Global DNA Methylation Modulation in Lipid Metabolism Disorders with a Marine-Derived Bioproduct
Source: Biology (Basel). 2023 Jun 2;12(6):806. doi: 10.3390/biology12060806 (PMC10295662; doi:10.3390/biology12060806)
Supplement: Supplementary file 1 [file biology-12-00806-s001.zip › Supplementary Table S1_biology-2302663_resub.pdf]

Supplementary Table S1. Demographics and clinical profiles of healthy patients before- (pre-) and one-month after (post-) treatment with RCI-1502 (750 mg/day)

Patient	Age (years)	Sex	Triglycerides (mg/dL) (pre)	Triglycerides (mg/dL) (post)	Glucose (mg/dL) (pre)	Glucose (mg/dL) (post)
1	35	F	57	63	92	85
2	50	M	323	286	92	99
3	54	F	61	78	81	77
4	56	M	178	120	105	109
5	46	F	157	153	95	99
6	45	F	55	62	94	94
7	28	F	52	46	85	86
8	39	F	103	61	88	95
9	43	M	153	99	84	84
10	55	F	116	131	101	95
11	54	M	48	48	99	103
